# The Neglected Role of Asphaltene in the Synthesis of Mesophase Pitch

**DOI:** 10.3390/molecules29071500

**Published:** 2024-03-27

**Authors:** Mingzhi Wang, Yulin Li, Haoyu Wang, Junjie Tao, Mingzhe Li, Yuzhu Shi, Xiaolong Zhou

**Affiliations:** International Joint Research Center of Green Energy Chemical Engineering, East China University of Science and Technology, Shanghai 200237, China; y15220008@mail.ecust.edu.cn (M.W.); a2663093157@163.com (Y.L.); a893748291@163.com (H.W.); 21010763@mail.ecust.edu.cn (J.T.); 13002928623@163.com (M.L.); Y15230015@mail.ecust.edu.cn (Y.S.)

**Keywords:** mesophase pitch, FCC slurry, molecular orientation, stability, temperature, pressure, reaction time, graphite-like microcrystals, asphaltene, softening point

## Abstract

This study investigates the synthesis of mesophase pitch using low-cost fluid catalytic cracking (FCC) slurry and waste fluid asphaltene (WFA) as raw materials through the co-carbonization method. The resulting mesophase pitch product and its formation mechanism were thoroughly analyzed. Various characterization techniques, including polarizing microscopy, softening point measurement, Fourier-transform infrared spectroscopy (FTIR), and thermogravimetric analysis (TGA), were employed to characterize and analyze the properties and structure of the mesophase pitch. The experimental results demonstrate that the optimal optical texture of the mesophase product is achieved under specific reaction conditions, including a temperature of 420 °C, pressure of 1 MPa, reaction time of 6 h, and the addition of 2% asphaltene. It was observed that a small amount of asphaltene contributes to the formation of mesophase pitch spheres, facilitating the development of the mesophase. However, excessive content of asphaltene may cover the surface of the mesophase spheres, impeding the contact between them and consequently compromising the optical texture of the mesophase pitch product. Furthermore, the inclusion of asphaltene promotes polymerization reactions in the system, leading to an increase in the average molecular weight of the mesophase pitch. Notably, when the amount of asphaltene added is 2%, the mesophase pitch demonstrates the lowest I_D_/I_G_ value, indicating superior molecular orientation and larger graphite-like microcrystals. Additionally, researchers found that at this asphaltene concentration, the mesophase pitch exhibits the highest degree of order, as evidenced by the maximum diffraction angle (2θ) and stacking height (Lc) values, and the minimum d_002_ value. Moreover, the addition of asphaltene enhances the yield and aromaticity of the mesophase pitch and significantly improves the thermal stability of the resulting product.

## 1. Introduction

In petroleum mixtures, the heavy fraction contains a significant amount of macromolecular non-hydrocarbon compounds, which are commonly classified as resins and asphaltenes. However, there is currently no internationally standardized definition or precise boundary for distinguishing between resins and asphaltenes [1,2,3]. The small molecules of n-alkanes that are insoluble in non-polar solvents but soluble in benzene are generally referred to as asphaltenes, representing the most polar and highest molecular weight non-hydrocarbon components in petroleum. The choice of organic solvent for asphaltene separation varies, and therefore, when presenting asphaltene content data, it is necessary to specify the solvent used, such as n-pentane asphaltene or n-heptane asphaltene. The amount of precipitated asphaltenes decreases with an increase in the relative molecular weight and solubility of n-alkanes in the asphaltene fraction. Resins, characterized by their relatively high polarity and molecular weight, are polydisperse macromolecular non-hydrocarbon compounds, ranking second only to asphaltenes in terms of polarity and molecular weight. However, there is no clear demarcation between resins and asphaltenes. Consequently, the reported data regarding resin content in petroleum may exhibit significant variations depending on the analysis methods employed. Chinese researchers have defined the components obtained by separating saturated and aromatic components from the n-heptane-soluble fraction of residual oil using alumina liquid chromatography as resins. In terms of appearance, resins appear as dark brown substances, typically existing as amorphous solids or viscous liquids. They melt at high temperatures and exhibit a relative density of approximately 1.0. Asphaltene, on the other hand, is a dark brown or black amorphous solid that does not melt when subjected to high temperatures. It is relatively brittle and prone to fracturing into pieces. The relative density of asphaltenes is also around 1.0, slightly higher than that of resins [4,5,6,7].

**Figure 1:**
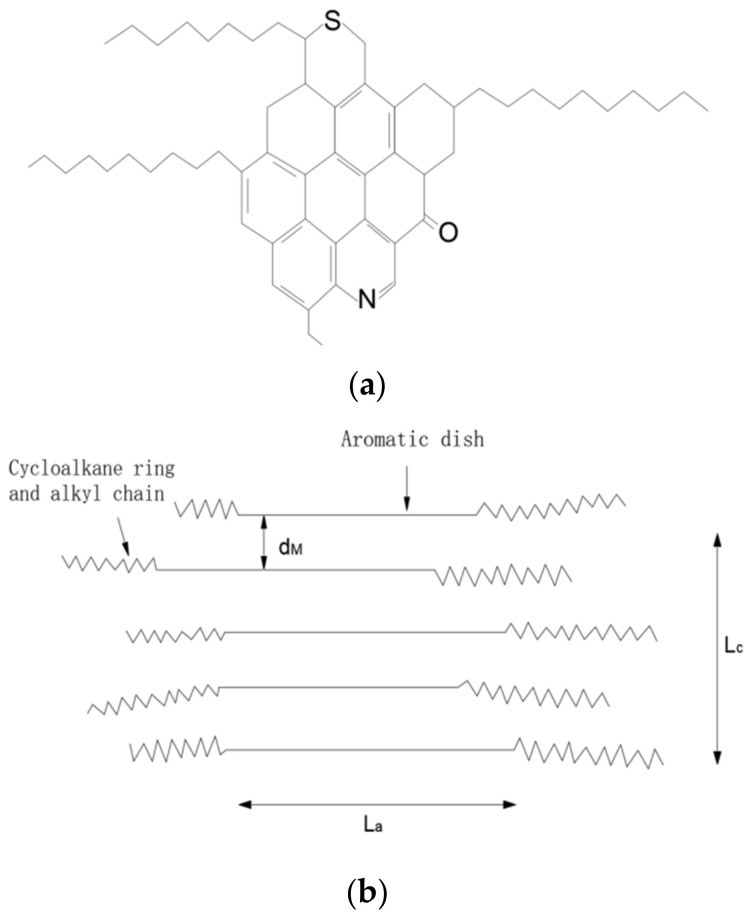

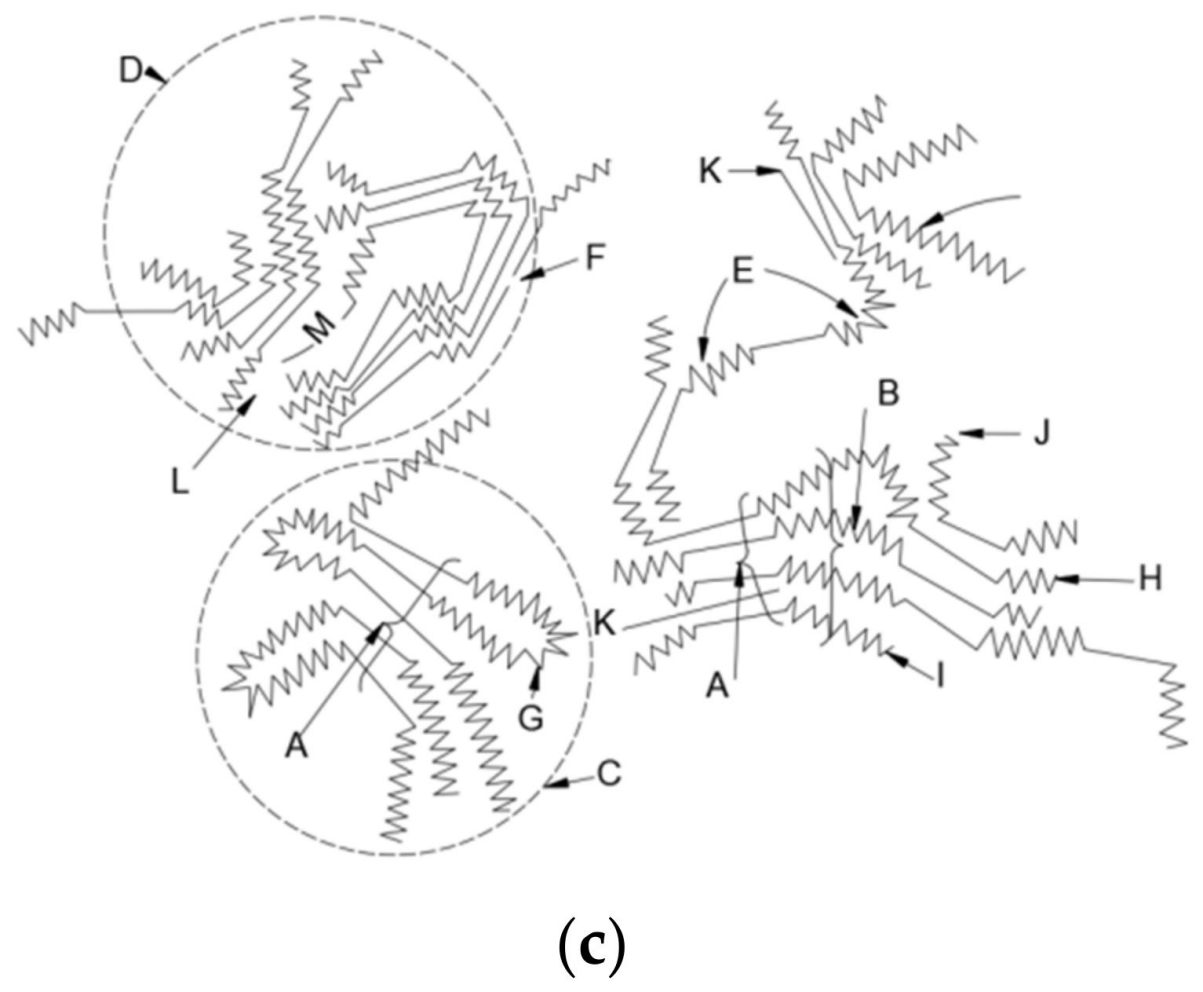
(**a**) Basic molecular structure of colloidal asphaltenaceous material; (**b**) asphaltene-like particles with a crystalline structure; (**c**) schematic diagram of the macroscopic structure of asphaltenes. The straight line indicates the aromatic ring system, and the zigzag line indicates the saturated structure (containing alkanes and naphthenes). A—grain; B—side chain bundle; C—a particle; D—a micelle; E—weak bond; F—one hole; G—intramolecular clusters; H—intermolecular cluster; I—gum; J—a single tablet; K—petroporphyrin; L—metal; M—insoluble component in micelle ; L_a_—average diameter; L_c_—stacking height; d_M_—interlayer spacing.

Due to the abundance of aromatic structures in asphaltene and resin, they exhibit relatively low hydrogen-to-carbon ratios, with asphaltene having a lower ratio compared to resin. The hydrogen-to-carbon ratio of resin is generally around 1.4, while for asphaltene, it ranges approximately between 1.1 and 1.3. Determining the average relative molecular weight of resin and asphaltene is a challenging task due to the presence of numerous heteroatoms in their molecular structures. The association between different molecules leads to the formation of supramolecular structures at various levels. Consequently, data obtained using different measurement methods can vary significantly. Therefore, it is crucial to specify the conditions and methods used when providing data on the average relative molecular weight of resin and asphaltene. Only data obtained using the same method under equivalent conditions can be considered comparable. The vapor pressure osmometry (VPO) method, which employs vapor pressure permeation, is commonly used to measure the average relative molecular weight [8,9,10]. The basic structure of resin and asphaltene molecules in petroleum consists of a dense aromatic ring system as the core, with multiple fused aromatic rings, surrounded by several cycloalkane rings. These aromatic and cycloalkane rings are attached to various length-variant normal or isomeric side chains. The molecular branches or ring systems may contain elements such as sulfur (S), nitrogen (N), and oxygen (O) and trace amounts of metal elements, as shown in Figure 1a [11,12,13]. This core structure, composed of a fused aromatic ring system, is the fundamental unit of resin and asphaltene molecules, also referred to as the unit structure or unit sheet. A resin or asphaltene molecule is composed of several such unit structures connected by methylene bridges of varying lengths, with oxygen or sulfur present between these bridge structures. Compared to resin, asphaltene has a higher average relative molecular weight and a higher level of aromatization. In terms of the number of rings per unit structure, asphaltene significantly exceeds resin. The resin unit structure contains approximately five aromatic rings, while the asphaltene unit structure contains around seven to ten aromatic rings. The aromatic rings in asphaltene primarily undergo peri-condensation, while in resin, both peri-condensation and kata-condensation occur between aromatic rings [14,15,16].

Graphite is a well-known hexagonal crystal structure composed of pure carbon. It consists of numerous overlapping layers of carbon atoms arranged in a network formation. Within each layer, the carbon atoms form regular hexagons, and the interlayer spacing is approximately 0.335 nm. The X-ray diffraction spectrum of graphite exhibits a sharp (002) peak at around 26°, indicating the ordered arrangement of carbon atom layers. In the early 1960s, T.F. Yen discovered that asphaltene, separated from petroleum, also exhibits a (002) peak at around 26° in its X-ray diffraction spectra. This suggests that asphaltene possesses a similar ordered structure to graphite. In other words, within asphaltene molecules, there are partially ordered crystalline structures formed by unit sheets with fused aromatic rings as the core. This ordering arises due to the overlap and synergistic effect of the π electron clouds of aromatic rings within and between the molecules. However, the presence of non-planar cycloalkanes and alkyl chains connected to these aromatic ring systems in asphaltene results in slightly larger interlayer spacing compared to graphite, approximately 0.36 nm. Figure 1b represents the crystalline structure particles of asphaltene [17,18,19,20]. Scientists also utilize the property of petroleum dispersion systems to determine the content of asphaltene by using a significant amount of low molecular weight n-alkanes to dilute crude oil or residue. By reducing the aromaticity and viscosity of the dispersion medium, the solvation layer of the gum in the micelles is disrupted, leading to the formation of larger aggregates of asphaltene, which then separate and precipitate into a distinct phase. The basic structure of asphaltene molecules is centered on a polycyclic aromatic ring system composed of multiple aromatic rings, surrounded by multiple cyclo-alkane rings, aromatic rings, and alkyl side chains. T.F. Yen proposed the model shown in Figure 1c to represent this structure [20,21,22,23]. However, in the production process of mesophase pitch, asphaltene is considered a by-product, and many researchers aim to minimize its content. However, if the content of asphaltene is too low, it is detrimental to the formation of mesophase pitch; but if the content of asphaltene is too high, it will destroy the optical texture of mesophase pitch, which is not conducive to being used as a precursor for producing carbon fibers; therefore, it is important to control the amount of asphaltene in the product. In our research, we plan to investigate the influence of asphaltene on the formation of mesophase pitch. Initially, our research group used FCC slurry to synthesize mesophase pitch [24], exploring optimal conditions such as temperature, pressure, and reaction time. Subsequently, asphaltene was employed as a co-carbonizing agent, and by varying the amount of asphaltene added, we continuously screened the reaction conditions to identify the production process of mesophase pitch with the best reaction efficiency. In this study, we incorporate WFA as an additive to analyze and discuss its impact on the structure, morphology, thermal properties, and optical texture of pyrolysis products. The objective of our research is to develop a co-carbonization agent that enhances the formation of high-quality mesophase pitch.

## 2. Results and Discussion

### 2.1. Determination of Optimal Reaction Conditions

#### 2.1.1. Determination of Reaction Temperature

Based on previous experimental results of the direct thermal condensation polymerization reaction of FCC-BL, the temperatures corresponding to the best product performance were found to be 400 °C and 420 °C. To validate the most suitable reaction temperature for the co-carbonization system, the research group opted to utilize asphaltene as the co-carbonizing agent with an addition amount of 1%, a system pressure of 1 MPa, and a reaction time of 6 h. The influence of different temperatures (390 °C, 400 °C, 410 °C, 420 °C, and 430 °C) on the co-carbonization method for preparing mesophase pitch was investigated, as depicted in Figure S3. In these micrographs, the corresponding part of the bright area is the anisotropic component, and the proportion of mesophase pitch products is determined by the ratio of anisotropic textures. From Figure S3, it is evident that different reaction temperatures have a substantial impact on the content and optical texture of the mesophase phase. At 390 °C and 400 °C, the content of the mesophase phase in the product is relatively low due to the reaction being a free radical reaction. At lower temperatures, the higher viscosity within the system hinders chain-initiated reactions, thereby significantly impeding the generation of mesophase phases. With increasing temperature, the proportion of the mesophase pitch shows an upward trend, accompanied by a transformation of the optical texture from a small flow structure to a large domain structure. At 420 °C, both the content of the mesophase pitch and the optical texture achieve optimal results. This is attributed to the elevated temperature intensifying the thermal reaction within the system, leading to abundant generation of free radicals from the decomposition of raw materials. This, in turn, facilitates further thermal cracking and condensation of the reaction. Simultaneously, the increased temperature reduces the viscosity of the reactants, promoting the condensation of aromatic hydrocarbons and the transfer of free radicals, resulting in the formation of a greater number of planar-fused ring macromolecules. These macromolecules arrange and stack freely, generating a significant quantity of mesophase pitch microspheres. These microspheres undergo fusion, growth, and rupture, ultimately forming a wide-ranging mesophase pitch asphalt. When the temperature exceeds 420 °C, the rates of thermal cracking and condensation reactions within the system increase significantly, leading to excessive carbonization of the intermediate products [25,26,27]. Consequently, the research group determined 420 °C as the optimal co-carbonization reaction temperature. An increase in temperature will increase the softening point of the product, but at the same time, it will reduce the yield of the product. Therefore, it is important to control the experimental temperature reasonably.

#### 2.1.2. Determination of Reaction Time

Based on the previous experimental findings of the direct thermal condensation reaction of FCC-BL, the optimal reaction time was observed to be 6 h at a reaction temperature of 420 °C. In order to explore the most suitable reaction time, the research team decided to employ 1% asphaltene as a co-carbonizing agent, with a system pressure of 1 MPa and a reaction temperature of 420 °C. The influence of different reaction times (4 h, 5 h, 6 h, 7 h, and 8 h) on the production of mesophase pitch using the co-carbonization method was investigated. As illustrated in Figure S4, when the reaction was conducted for 4 h, the proportion of mesophase pitch in the resulting product was relatively low. However, after 5 h of reaction, there was a noticeable increase in the proportion of mesophase pitch. Upon reaching 6 h of reaction time, the proportion of mesophase pitch exceeded 90%, with the optical texture predominantly exhibiting a large domain structure. When the reaction time exceeds 6 h, the optical texture of the mesophase pitch in the product becomes disordered, and the domain structure significantly deteriorates. The reason is that the system contains a large number of aromatic structures and chain alkanes, which have high reactivity and greatly promote thermal cracking reactions. Consequently, the content of aromatic free radicals is considerably enhanced, thereby facilitating the abundant generation of planar-fused ring aromatic molecules. These larger molecules undergo directed arrangement and stacking, ultimately giving rise to the formation of mesophase microspheres. These microspheres interact, merge, grow, and eventually rupture, leading to the formation of sheet-like mesophase pitch, which exhibits a flow structure basin-like morphology under the influence of pressure. However, when the reaction time surpasses the critical threshold, excessive aggregation of intermediate molecules occurs, and the stacking between molecules tends to assume a disordered state, ultimately resulting in carbonization [28,29,30]. In light of the aforementioned observations, the research team established a reaction time of 6 h for their study.

#### 2.1.3. Determination of Reaction Pressure

Based on prior experimental results of the direct thermal polycondensation reaction of FCC-BL, the system pressure yielding the best experimental outcome was approximately 1 or 2 MPa. To explore the most suitable pressure for the co-carbonization reaction, the research team employed asphaltene as a co-carbonization agent with a 1% addition rate, a reaction temperature of 420 °C, and a reaction time of 6 h. The impact of different reaction pressures (0, 0.5 MPa, 1 MPa, 1.5 MPa, and 2 MPa) on the production of mesophase pitch using the co-carbonization method was investigated. As shown in Figure S5, when the system pressure is low, a large number of light components will escape from the reaction system, causing a significant increase in the viscosity between the raw materials, greatly increasing the degree of polycondensation of the product molecules. The large product molecules have a serious impact on the optical texture, while the escaping light component substances inhibit the growth of mesophase pitch, making it tend to be disordered, which is manifested as a mosaic structure in the product. As the pressure gradually increases, the morphology of mesophase pitch gradually changes from a mosaic structure to a large-domain structure. When the pressure is 1.0 MPa, the optical texture of the product is the best, approaching 100%. However, when the pressure exceeds 1.5 MPa, the optical texture deteriorates significantly. This is due to the suppression of light component escape at pressures beyond the critical threshold, hindering the ordered accumulation of condensed ring macromolecules and the fusion and growth of mesophase pitch microspheres, ultimately disrupting the ordered structure of the mesophase pitch product. Consequently, the optical texture of the mesophase pitch product deteriorates. When the pressure reaches 2.0 MPa, excessive pressure impedes the coalescence of mesophase microspheres, resulting in incomplete development of mesophase pitch [31,32]. Thus, the research team determined a reaction pressure of 1.0 MPa.

The properties of the modified pitch were analyzed and are presented in Table 1. The hydrogen–carbon ratio of the product gradually decreases as the proportion of WFA increases, indicating a transformation of aliphatic structures to aromatic ring structures. The content of toluene insoluble substance (TI) does not show a discernible pattern of change, while the content of quinoline insoluble substance (QI) increases progressively. This can be attributed to the promotion of polymerization reactions within the system with increasing WFA, resulting in the formation of more quinoline-insoluble substances. The yield and softening point of the product exhibits significant improvements, which can be attributed to two factors: firstly, both the carbonization rate of the pitch generated by WFA and FCC thermal condensation increase, and secondly, the cooperative effect of WFA and FCC thermal condensation during the co-carbonization process leads to an overall increase in the softening point and yield of the product. Notably, the ash content remains unchanged throughout the entire co-carbonization process [33].

### 2.2. Analysis of Mesophase’s Crystal Structure

**Figure 2:**
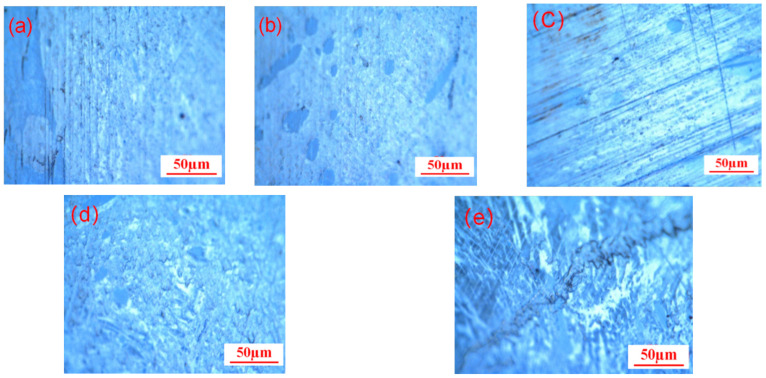
Optical texture picture of modified mesophase pitch with different WFA content. (**a**) 0%; (**b**) 1%; (**c**) 2%; (**d**) 3%; (**e**) 5%.

The characterization of mesophase pitch’s microcrystalline size and molecular orientation is commonly performed using X-ray diffraction and Raman spectroscopy. Initially, the optical textures of five sample groups (MMP-0, MMP-1, MMP-2, MMP-3, and MMP-5) were examined. The specific reference standards for the optical texture of the product can be found in Table S2. Figure 2 illustrates the optical textures of the five types of mesophase pitch. From the diagram, it is evident that the presence of anisotropic components is relatively low when WFA is not added. However, the addition of WFA to the reaction system significantly influences the optical microstructure of mesophase pitch. Upon the initial addition of WFA to the reaction system, mesophase pitch gradually exhibits a large domain and extensively streamlined optical texture. When the WFA addition reaches 2%, the content of mesophase in the large domain streamlined structure reaches its maximum, approximately 95%. However, as the WFA content increases further, the content of the wide-area streamlined mesophase decreases gradually, and a distinct mosaic-like structure starts to appear on the surface. When the WFA addition reaches 5%, the optical texture of the mesophase pitch surface becomes severely damaged, with the presence of anisotropic components almost indiscernible. The surface transforms into a pronounced mosaic form. Based on this analysis, it can be inferred that the addition of WFA significantly influences the nucleation, growth, and aggregation of mesophase microspheres. Previous studies have indicated [34] that WFA acts as a nucleating agent to facilitate the formation of mesophase microspheres. When a small amount of WFA is added to the system, the number of nucleation sites in the system decreases, and the number of sites where the active molecules undergo π-π conjugation and adhere also decreases. Consequently, the system contains a higher proportion of highly mobile active molecules, promoting the growth and aggregation of mesophase microspheres. However, excessive WFA content leads to its accumulation on the surface of mesophase spheres, impeding their proximity and fusion, thereby creating a mosaic-like structure. Ultimately, this causes severe damage to the optical texture of mesophase pitch’s surface.

**Figure 3:**
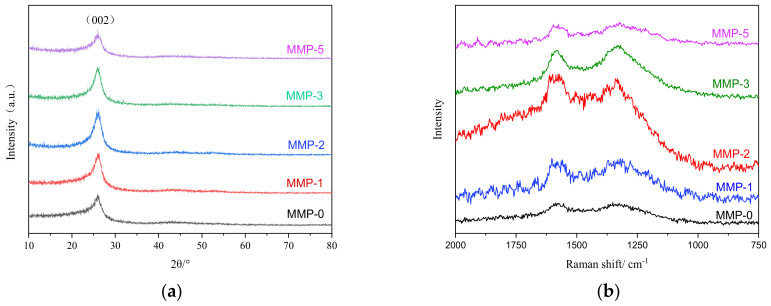
(**a**) XRD and (**b**) Raman patterns of the modified mesophase pitch.

Figure 3 presents the XRD and Raman spectra of five types of mesophase pitch [35,36,37,38,39]. The microcrystalline size parameters of the mesophase pitch, calculated using the Bragg equation and Scheler equation in conjunction with these two characterization methods, are provided in Table 2. From Figure 3a, it is evident that all five mesophase pitches exhibit high-intensity carbon (002) diffraction peaks around 2θ = 25°. This indicates that these asphalts possess a significant degree of molecular orientation. The intensity order of the carbon (002) surface diffraction peaks for the five mesophase pitch is as follows: MMP-0 < MMP-1 < MMP-5 < MMP-3 < MMP-2, with the corresponding 2θ values following the order: MMP-0 < MMP-1 < MMP-5 < MMP-3 < MMP-2. Furthermore, by employing the Bragg equation and Scheler equation, the stacking height (Lc) and molecular spacing (d_002_) of the molecular layers were calculated. As the 2θ value increases, the corresponding Lc value increases while the d_002_ value decreases, indicating a higher level of orderliness in the product. Table 2 reveals that with an increase in the proportion of WFA, the (002) diffraction peak initially shifts towards higher angles and then towards lower angles. This suggests that the structure of the mesophase pitch first tends towards orderliness and subsequently transitions to disorderliness. Notably, MMP-2 exhibits the highest 2θ value, indicating that when the WFA addition reaches 2%, the resulting mesophase pitch attains the highest degree of orderliness. To summarize, the content of WFA can alter the regularity of the carbon layer in pyrolysis products. What is more, the orderliness of mesophase pitch products primarily depends on their distinct molecular chemical compositions and molecular configurations.

The inclusion of WFA in the system introduces a significant number of aromatic rings and cycloalkanes. A small amount of WFA addition promotes the development of the mesophase pitch and increases its content. However, the excessive addition of WFA leads to an increased number of aromatic cores in polycyclic aromatic hydrocarbons, resulting in a higher quantity of aromatic nuclei. The structural variations among different aromatic nuclei reduce the conjugation of the mesophase pitch molecules, leading to inconsistent core orientations. Consequently, the planarity of the mesophase pitch molecules decreases, resulting in an increase in intermolecular interlayer spacing (d_002_). On the other hand, despite the unfavorable effect of different aromatic core orientations on molecular stacking, the molecular structure of WFA contains a substantial number of short-chain alkanes. These alkanes contribute to enhancing the mobility of MMP molecules, thereby promoting an increase in the number of molecular stacking layers and the molecular stacking height. This observation aligns with recent research findings [37]. The presence of higher stacking layers and heights in the mesophase pitch products is also closely associated with the introduction of certain short-chain alkanes during the co-carbonization process.

The Raman spectrum of carbon materials exhibits two prominent peaks [37,38,39] within the primary spectral range of 1000~2000 cm^−1^, namely the G peak and the D peak. The D peak, located around 1360 cm^−1^, is attributed to lattice defects in graphite, the low symmetry of carbon structures, and the disordered arrangement of edges. An increase in structural disorder and a decrease in microcrystalline size contribute to the enhanced intensity of the D peak. The G peak is located near 1580 cm^−1^. For mesophase pitch, the greater the intensity of the D peak, the higher its disorder, and the greater the intensity of the G peak, the higher its order. The relative intensity ratio of the D and G peaks, denoted as I_D_/I_G_, reflects the quality of molecular orientation and structure. A lower I_D_/I_G_ value suggests better molecular orientation and larger graphite-like microcrystals. According to Table 3, as the proportion of WFA increases, the I_D_/I_G_ values of the mesophase pitch products initially decrease and then increase. When the WFA addition reaches 2%, the I_D_/I_G_ value is minimized, indicating that the ordered structure of the pyrolysis products first increases and then decreases. Notably, MMP-2 exhibits the highest degree of structural order, aligning with the findings from XRD analysis. In conclusion, the appropriate addition of WFA promotes an increase in the orderliness of the mesophase pitch in the product. However, excessive amounts of WFA may lead to an elevation in the disorder of the mesophase pitch.

### 2.3. Molecular Structure Analysis of Mesophase Pitch

**Figure 4:**
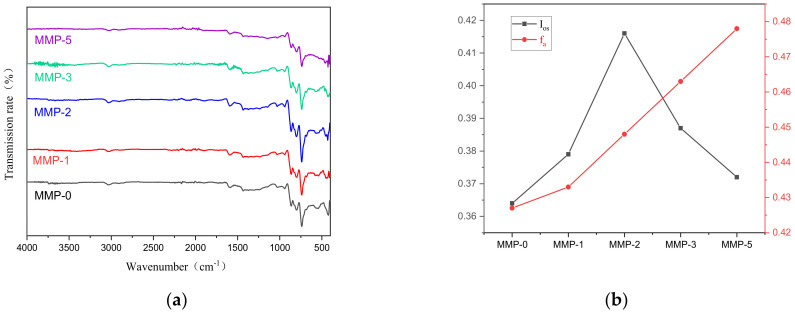
(**a**) FTIR spectra and (**b**) FTIR structural index parameters of different modified mesophase pitches.

Fourier-transform infrared (FT-IR) spectroscopy is a commonly used method to provide functional group information in complex solid materials [40,41,42]; specific reference standards can be found in Table S3. In the FT-IR spectrum, specific peaks can be assigned to various functional groups present in the sample. For instance, the peak around 3040 cm^−1^ corresponds to the stretching vibration absorption of aromatic C-H bonds, while the peak near 2950 cm^−1^ is attributed to the methyl absorption peak of fatty hydrocarbons. Peaks within the range of 2930–2700 cm^−1^ represent the stretching vibration absorption of C-H bonds in fatty hydrocarbons. The absorption peak at 1600 cm^−1^ is associated with the stretching vibration of the aromatic skeleton, whereas the peak at 1450 cm^−1^ corresponds to the bending vibration absorption of methylene groups in cycloalkane structures and fatty branch chains. The absorption peak at 1380 cm^−1^ indicates the bending vibration of -CH_3_ groups on the benzene ring, and the peaks at 880–750 cm^−1^ represent out-of-plane bending vibrations of aromatic C-H bonds. Notably, the peak at 880 cm^−1^ corresponds to an isolated out-of-plane bending vibration of aromatic C-H, while the peak at 750 cm^−1^ arises from the synergistic effect of four adjacent aromatic C-H bonds. The peaks within the range of 500–460 cm^−1^ gradually decrease, and the corresponding characteristic peaks in this range are caused by the outward bending vibration of the C-H plane of aromatic rings and substituted aromatic rings containing four adjacent hydrogens. From Figure 4a, it can be seen that before the addition of WFA, the content of aliphatic side chains in the mesophase pitch is slightly higher than that after the addition of WFA. Furthermore, with increasing amounts of WFA, the absorption peak of fatty branch chains in the mesophase pitch shows minimal variation, whereas the absorption peak of aromatic groups initially weakens and then strengthens. Upon analyzing the FT-IR spectrum, it could be observed that MMP consists of planar aromatic molecules with alkyl chain substituents on the aromatic ring. The ortho-substitution index (I_os_), which indicates the relative size of aromatic molecules, can be calculated using Formula (2). The aromaticity (fa) of mesophase pitch can be determined using Formula 3, where A represents the absorption intensity of a specific peak. According to Figure 4b, as the proportion of WFA increases, the ortho-substitution index (I_os_) of ortho-substituted aromatic rings initially increases and then decreases. Simultaneously, the fragrance of the product also increases with the addition of WFA. Based on these findings, it can be inferred that a small amount of WFA added to the reaction system can promote the dehydrogenation condensation reaction of small molecule aromatic rings, thereby facilitating the aromatization reaction. However, excessive amounts of WFA may restrict the accumulation of smaller planar aromatic molecules and inhibit the formation of larger planar bitter aromatic molecules.

**Figure 5:**
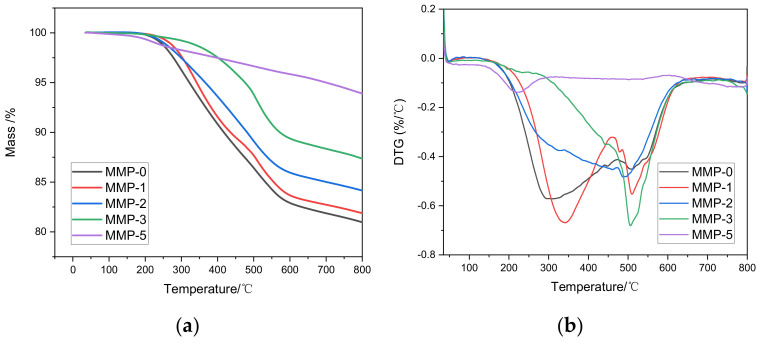
(**a**) TG and (**b**) DTA results of the different modified mesophase pitches.

The graphs displayed in Figure 5 illustrate the thermogravimetric (TG) and derivative thermogravimetric (DTG) curves of mesophase pitch. From the figure, it can be seen that the pyrolysis of the product is divided into two stages. The first stage is between 200–570 °C, and the five types of mesophase pitch exhibit weight loss starting at approximately 200 °C. This reduction in sample mass primarily stems from the evaporation of light components and volatile substances. As the temperature reaches 380 °C, the sample experiences a continual decrease in mass due to molecular dissociation and dehydrogenation. The second stage is at 570–800 °C. Low molecular weight gases (various alkanes) are mainly produced through side chain cracking reactions, polymerization reactions, and aromatization reactions, and the weight loss curves of the five mesophase pitches begin to stabilize. In the absence of WFA in the reaction system, the carbon yield of mesophase pitch is minimal. However, with an increasing proportion of WFA, the carbon yield of mesophase pitch gradually rises. This observation suggests that WFA can enhance the thermal stability of mesophase pitch. The reason for this phenomenon is that the addition of WFA can effectively promote the thermal polymerization reaction. Notably, when the WFA ratio reaches 5%, the carbon yield can reach a substantial value of up to 93%. These findings provide evidence that an appropriate amount of WFA can stimulate thermal polymerization reactions [43,44]. For applications requiring improved thermal stability of the reaction system, it is advisable to explore suitable co-carbonizing agents that facilitate the transformation of small molecules into planar molecular structures during thermal decomposition.

**Figure 6:**
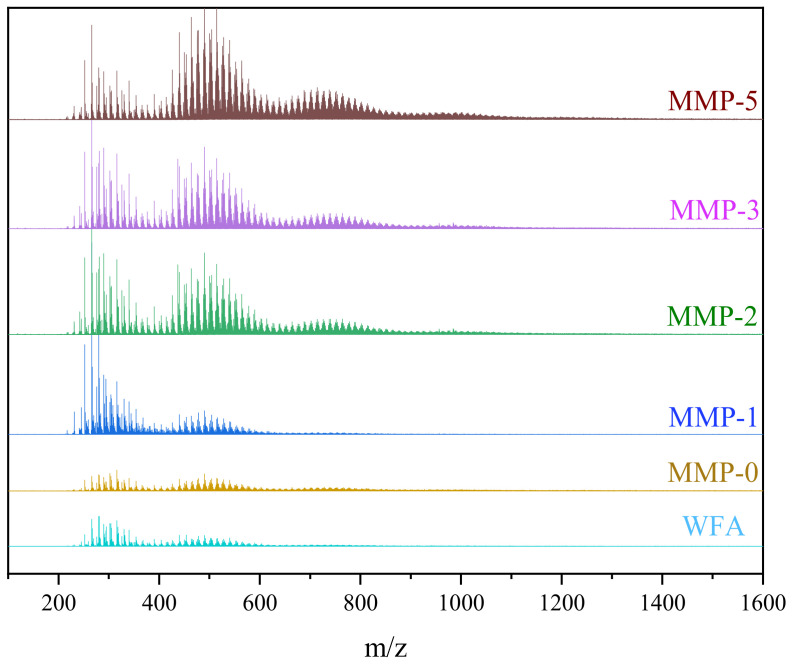
MALDI-TOF MS spectra of the different modified mesophase pitches.

The MALDI-MS spectrum presented in Figure 6 exhibits a broad distribution of molecular weights for mesophase pitch, ranging from 100 to 3000 *m*/*z*. The average molecular weights of asphaltene, MMP-0, MMP-1, MMP-2, MMP-3, and MMP-5 are determined to be 343, 392, 446, 540, 587, and 612, respectively. The fluctuations in average molecular weight suggest that the composition of the five mesophase pitches comprises polycyclic aromatic hydrocarbon molecules with different numbers of units, typically characterized by 2–4 hexagonal rings. Polycyclic aromatic hydrocarbon molecules can be categorized into monomers (*m*/*z* ≈ 200–400), dimers (*m*/*z* ≈ 400–700), trimers (*m*/*z* ≈ 700–1000), and tetramers (*m*/*z* ≈ 1000–1200) based on the number of aromatic units. In the absence of WFA in the system, mesophase pitch predominantly consists of monomers, resulting in a lower average molecular weight. This phenomenon can be attributed to the dealkylation reaction during thermal polymerization, leading to a reduction in molecular weight. Additionally, the dealkylation reaction generates a significant number of free radicals, causing a rapid increase in system viscosity, which hinders the progress of the polymerization reaction. However, with the introduction of WFA, the average molecular weight of mesophase pitch gradually increases. This effect arises from the synergistic interaction between the two raw materials during co-carbonization. Specifically, the abundant cycloalkane structure in the molecular composition of WFA reduces the dealkylation reaction rate during FCC-BL pyrolysis. Moreover, it collaborates with the rich concentration of short fatty hydrocarbon components in FCC-BL, collectively reducing the viscosity of the reaction system. Consequently, this facilitates the polymerization reaction of the raw material molecules during the thermal treatment process. Based on the aforementioned details, it can be deduced that the components generated from WFA pyrolysis promote the formation of large polycyclic aromatic molecules through the cross-linking of aromatic units [45,46]. As a result, the molecular weight of these anisotropic components progressively increases with the augmentation of WFA addition.

### 2.4. ^1^H-NMR Analysis and ^13^C-NMR Analysis

^1^H-NMR analysis and ^13^C-NMR analysis are indispensable techniques employed for determining the relative proportions of hydrogen and carbon in the aliphatic and aromatic constituents of diverse mesophase samples [47,48]. Specific reference standards can be found in Table S4. Table 3 provides a comprehensive summary of the hydrogen and carbon atom contents in intermediate-phase pitch samples prepared using varying amounts of WFA as a co-carbonizing agent. The aliphatic protons encompass H_α_, H_β_, and H_r_ while the aromatic protons encompass monoaromatic, biaromatic, and polyaromatic protons. The chemical shifts of aromatic protons in ^1^H-NMR spectroscopy lie within the range of 6.0 to 9.0 ppm. Among these, monoaromatic protons (H_ar(mono)_) exhibit chemical shifts in the range of 6.0 to 7.1 ppm, biaromatic protons (H_ar(di)_) exhibit chemical shifts in the range of 7.1 to 8.2 ppm, and polyaromatic protons (H_ar(poly)_) exhibit chemical shifts in the range of 8.2 to 9.0 ppm. The chemical shifts of aliphatic protons (H_al_) predominantly concentrate in the range of 0.5 to 4.5 ppm. Specifically, the chemical shift of H_α_ lies within the range of 2.1 to 4.5 ppm, that of H_β_ lies within 1.1 to 2.1 ppm, and that of H_r_ lies within 0.5 to 1.1 ppm. In ^13^C-NMR spectroscopy, the chemical shift of saturated carbon (C_sat_) is observed at 5 to 50 ppm, while aromatic carbon (C_ar_) exhibits a chemical shift of 100 to 160 ppm.

The data of the ^1^H-NMR spectrum reveal that the addition of WFA leads to an increase in the ratio of H_ar_ and a decrease in the ratio of H_al_. This observation suggests that the co-carbonization reaction facilitates the conversion of linear aliphatic chains into bridged structures. The predominant reaction in this co-carbonization process is the cyclization reaction, which involves a dehydrogenation polymerization step that generates hydrogenated aromatic hydrocarbon structures. These structures subsequently undergo rapid conversion into aromatic hydrocarbons. In the reaction system, if the content of H_α_ is higher than that of H_β_ and H_r_, this indicates that the majority of alkyl groups are connected to the aromatic ring in the form of methylene. The presence of alkyl groups promotes catalytic cracking and the formation of more aromatic hydrocarbons, thereby enhancing the performance of the mesophase pitch. With increasing WFA content, the proportion of H_β_ and H_r_ decreases, primarily due to the occurrence of cracking reactions at the β and r positions of the fatty chain under high-temperature conditions. Similarly, an increase in the hydrogen aromaticity index signifies the higher content of aromatic hydrogen in the prepared mesophase structure. The ^13^C-NMR data also demonstrate that the mesophase pitch prepared with WFA exhibits higher content of aromatic carbon compared to the pitch prepared without WFA.

The data presented in Table 3 demonstrate that the carbon aromaticity (C_ar_/C_sat_) is higher compared to the hydrogen aromaticity (H_ar_/H_al_), suggesting that the formation of condensed aromatic structures involves dehydrogenation, condensation, and polymerization reactions. In conclusion, the condensation and polymerization reactions occurring during the co-carbonization process entail the removal of hydrogen, leading to the generation of high molecular weight aromatic polymers.

### 2.5. Morphology and Structure of Final Co-Carbonization Products at High Temperature (SEM)

**Figure 7:**
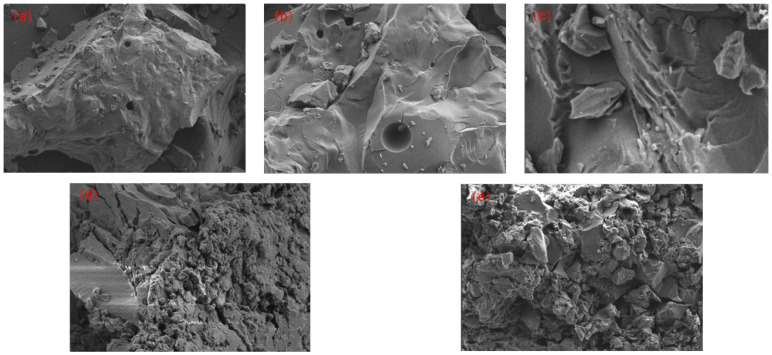
SEM images of different modified mesophase pitches with different WFA content. (**a**) 0%; (**b**) 1%; (**c**) 2%; (**d**) 3%; (**e**) 5%.

The SEM images in Figure 7 depict the morphology and structure of the pyrolysis products obtained from FCC-BL with varying amounts of added WFA. Figure 7a reveals that in the absence of WFA, the surface of the product mesophase pitch exhibits numerous granular impurities. At this time, the granular impurities mainly include unmelted intermediate phases and quinoline-insoluble substances. However, in Figure 7b, the addition of 1% WFA leads to a significant reduction in the number of granular impurities and the development of a small flow structure is noticeably improved compared to the system without WFA. Figure 7c demonstrates that with a WFA addition of 2%, almost all the granular impurities on the pyrolysis product surface disappear, resulting in a complete layered structure. On the other hand, Figure 7d shows that at WFA content of 3%, substantial accumulation of impurities occurs on the pyrolysis product surface and the main component of impurities at this time is quinoline insoluble substances, leading to a significant impact on the layered structure. Finally, in Figure 7e, it is evident that when the WFA content reaches 5%, the pyrolysis product surface is nearly entirely covered by impurities, resulting in the almost complete disappearance or coverage of the layered structure [49,50,51,52].

In conclusion, the observed variations in microstructure are strongly correlated with the pyrolysis and carbonization processes. The addition of WFA facilitates the nucleation and growth of the mesophase pitch, thereby enhancing aromaticity. However, when the WFA content surpasses a critical threshold, it has the potential to aggregate on the surface of pyrolysis products or mesophase microspheres, impeding their mutual approach and fusion. This phenomenon ultimately leads to the formation of a mosaic structure.

### 2.6. Experimental Mechanism

**Figure 8:**
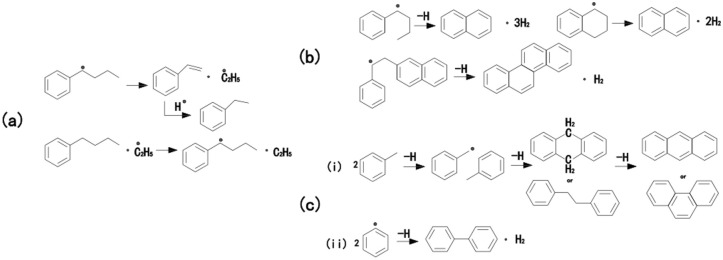
(**a**) The preliminary thermal decomposition reaction; (**b**) intramolecular condensation reaction; (**c**) condensation reaction between adjacent molecules. This includes: (**i**) Condensation reaction between adjacent molecules involving alkyl side chains. (**ii**)Thermal condensation reaction between adjacent molecules involving aromatic nuclei.

In general, the thermal decomposition of polycyclic aromatic hydrocarbons in the liquid phase can be divided into two stages: an initial thermal decomposition reaction and a subsequent thermal polymerization reaction. Peng et al. [53], in their study of n-butyl benzene under high-pressure conditions, found that styrene was the main product during the early stage of the reaction. The formation of styrene was attributed to the cleavage of the C_2_-C_3_ bond in the alkyl substituent, followed by rapid capture of hydrogen radicals to form styrene ethane, as depicted in Figure 8a. Savage investigated the pyrolysis mechanism of dodecyl pyrene and observed that the formation of the major product, ethyl pyrene, occurred through the removal of α-H free radicals at the C position by n-decane radicals, leading to the formation of α-C free radicals. Subsequently, the α-C free radicals underwent C_2_-C_3_ bond cleavage to yield vinyl pyrene. Based on their research, the dissociation energies were determined to be 375 kJ/mol, 428.4 kJ/mol, and 458.2 kJ/mol for benzyl, alkyl, and phenyl groups, respectively. This conclusion suggests that benzyl radicals, namely α-C free radicals, are more readily formed in aromatic compounds. Thus, it is widely accepted that the formation of α-C free radicals on alkyl side chains plays a significant role in the decomposition reactions of aromatic compounds containing long alkyl chains.

The main reaction of polycyclic aromatic hydrocarbons under medium temperature liquid phase cracking reaction conditions is condensation reaction. Based on the molecular structure of the reactants involved in the condensation reaction, condensation reactions can be roughly divided into the following types [54,55]: (1) intramolecular condensation reaction; for example, the conversion of n-butyl benzene to naphthalene or the formation of polycyclic aromatic hydrocarbons generally occurs on the -CH_2_-CH_2_- chain between two aromatic rings, as shown in Figure 8b. (2) Intermolecular condensation reactions; the intermolecular condensation reaction includes: the condensation reaction between adjacent molecules of alkyl side chains, as well as the thermal condensation reaction between adjacent molecules of aromatic nuclei, as shown in Figure 8c. The above different types of condensation reactions can be carried out in different ionization forms to obtain higher molecular weight polycyclic aromatic hydrocarbons.

The reaction mechanism employed in this experiment exhibits similarities to the Figure 8a mechanism in the initial stages, involving the generation of α-C free radicals on aromatic compounds within the system. In the later stages, the mechanism resembles the Figure 8b,c mechanisms, whereby the alkane moieties attached to the aromatic nuclei in aromatic compounds approach each other. During the polymerization process, these moieties, owing to their high electronegativity, engage in intramolecular or intermolecular cross-coupling reactions, leading to the formation of additional aromatic ring-containing molecules. This observation aligns with the conclusions drawn from the FT-IR characterization mentioned earlier.

The co-carbonizing agent asphaltene contains rich cyclic alkyl structures and long alkyl side chains, significantly reducing the viscosity of the reaction system, improving the flow-ability of the raw materials, increasing the probability of contact between raw material molecules, promoting the ordered stacking of planar aromatic macromolecules, and the subsequent fusion and growth of mesophase pitch microsphere, ultimately forming a large domain texture of mesophase pitch; In addition, asphaltene exhibits excellent reactivity during the reaction process, promoting the thermal reaction of the raw materials, leading to the generation of more free radicals in the reaction system, promoting the condensation of aromatic molecules, and facilitating the formation of mesophase pitch.

## 3. Experiment

### 3.1. Determination of Element Content

The carbon, hydrogen, nitrogen, and sulfur elements in the sample were quantified using a German elemental analyzer (Elementar vario EL III). The analyzer employs the difference subtraction method to calculate the oxygen element content.

### 3.2. Determination of Ash Content

We determined the ash content of the sample. We weighed the clean porcelain crucible and recorded it as m_0_. Then, we placed 2.5 g of the sample into a crucible and weighed it, denoted as m_1_. We rolled the quantitative filter paper into a conical shape, cut a section about 5 mm away from the tip, and then placed it into the crucible as the ignition core. After soaking the filter paper in the sample, we ignited and burned it until the sample stopped burning and there was no smoke. There was a shiny black substance remaining in the crucible, which is called residual carbon. We placed the crucible with the residue in a muffle furnace and burned it at 775 ± 25 °C for 1.5–2 h until the residue inside the crucible completely burned into white or grayish white powder. We cooled the crucible in air for 3 min before placing it in a dryer and weighing it to a constant weight, recorded as m_2_. The ash content of the sample was calculated by Formula (1):

(1) $$Ash\;content=\frac{m_{2}-m_{0}}{m_{1}-m_{0}}\times 100\%$$

### 3.3. Determination of Four Components

The four components of the oil slurry were determined using the IATROSCAN MK-6s TLC-FID thin-layer chromatograph from Shanghai branch of Japan’s Yatelon Company.. A microinjector was used to point the sample on the chromatographic rod. The three developing agents were n-hexane, toluene, dichloromethane, and methanol (volume ratio 95:5), with solvent heights of 100 mm, 55 mm, and 25 mm, respectively. After unfolding the solution on the chromatographic rod, we placed the rod in a thin-layer chromatograph for detection and used the software provided by TLC-FID to analyze and calculate the content of aromatics, saturates, resins, and asphaltenes.

### 3.4. Infrared Spectroscopy Analysis (FT-IR)

The sample was subjected to infrared spectroscopy analysis using a Nicolet Magana IR500 infrared spectrometer (NICOLET Company, Wisconsin, USA). This technique provided information about the types of functional groups present in the test sample. The sample was compressed with KBr to form a pellet, and the spectral scanning range was set at 400–4000 cm^−1^. The ortho-substitution index (I_os_), which represents the relative size of aromatic molecules, was calculated according to Formula (2). The aromaticity (fa) of the mesophase pitches was determined using Formula (3):

(2) $$I_{os}=\frac{{Ab}_{750}}{{Ab}_{750}+{Ab}_{815}+{Ab}_{880}}$$

(3) $$fa=\frac{{0.574Ab}_{1600}}{{Ab}_{1600}+{0.16Ab}_{1460}+{0.23Ab}_{1330}}+0.024$$

Among them, Ab_750_, Ab_815_, Ab_880_, Ab_1330_, Ab_1460_, and Ab_1600_ are the absorption intensities of the absorption peaks at 750, 815, 880, 1330, and 1460 cm^−1^, respectively.

### 3.5. Nuclear Magnetic Resonance Hydrogen Spectroscopy (^1^H-NMR) and Carbon Spectroscopy (^13^C-NMR) Analysis

Nuclear magnetic resonance (NMR) analysis of asphaltene and its co-carbonization products is an important analytical method. In NMR analysis, the chemical shifts of carbon and hydrogen atoms in different environments and positions vary. NMR provides clear structural parameters of asphaltene, allowing for the deduction of its molecular structure. The B-L method, based on hydrogen NMR spectroscopy, is used to calculate the structural parameters of asphaltene. The instrument utilized in this section is the Ascend 600 nuclear magnetic resonance spectrometer from Beijing branch of Switzerland’s Bruker Company. Deuterated chloroform (CDCl_3_) served as the deuterated solvent. The sample was prepared by dissolving 100 mg of the sample in 2.0 mL of deuterated chloroform. The hydrogen spectrum was scanned 128 times, while the carbon spectrum was scanned 512 times. An AVANCE III 400 MHz nuclear magnetic resonance spectrometer(Bruker Company, Switzerland) was employed for NMR analysis of the sample, providing information on the sample’s structural composition. Deuterated chloroform was used as the solvent, and the B-L method was employed for structural analysis.

### 3.6. Determination of Softening Point

The determination of the softening point follows the global method outlined in the GB/T4507-2014 standard. Methyl silicone oil was chosen as the heating medium. The sample was initially ground and then heated until it reached a flowable state. Simultaneously, the copper ring was preheated and placed on a flat metal plate. The sample was then poured into two copper rings, slightly above the plane of the copper ring. After solidification, any excess sample was scraped off from the top of the copper ring to ensure a smooth surface. The copper ring was positioned for testing, and each sample underwent two parallel experiments. The final experimental results were averaged. If the sample’s softening point exceeds 180 °C, the DMA Q800 dynamic mechanical thermal analyzer can be used for measurement.

### 3.7. Determination of Quinoline Insoluble Content and Toluene Insoluble Substance

The sample was pulverized and subjected to a drying process at 60 °C for 12 h. A quantity of 1 g of the sample was carefully weighed and enclosed in filter paper, followed by placement in a Soxhlet extractor. Initially, extraction was performed using toluene as the solvent at a temperature of 130 °C. Extraction continued with quinoline as the solvent at 110 °C until the reflux solution within the extractor became colorless, indicating completion of the extraction process. Subsequently, the solvent was eliminated from the extracted solution through distillation. The resulting mixture, together with the filter paper, was introduced into a vacuum drying oven and subjected to a drying period of 2 h at 100 °C. Each component was then accurately weighed, leading to the determination of the final content. The processing method for measuring and calculating toluene insoluble matter is as follows: Following the aforementioned treatment method, place the mesophase pitch sample in a filter paper cylinder, and then place the filter paper cylinder in a Soxhlet extraction apparatus and extract with toluene. After extraction is complete, the remaining pitch in the filter paper cylinder is the toluene-insoluble component, and parallel samples were measured for each group of samples, and the average value was taken.

### 3.8. Polarization Microscope Characterization

The optical texture of the sample was examined using an LV100N POL polarizing microscope, manufactured by the Nikon Corporation of Tokyo, Japan. The process involved mixing epoxy resin A adhesive and B adhesive in a specific ratio, which was then poured into a mold and combined with the sample before being subjected to heat treatment for solidification. The polishing procedure was conducted using an MP-2B fully automated polishing machine, produced by Fujian Testing Instrument and Equipment (Xiamen, China) Co., Ltd. Initially, polishing was performed using 300–5000 mesh sandpaper, followed by a polishing step with 10,000 mesh polishing paste to achieve a mirror-like finish on the specimen. Subsequently, the optical texture of the sample was examined under the polarization microscope at various magnifications. Among them, the area corresponding to the reflection and brightness of the sample under microscope observation is the anisotropic textures, which is the mesophase pitch; the dark areas are disordered isotropic asphalt. We imported the captured optical morphology image into ImageJ software and calculated the number of mesophase pitch pixels and the approximate proportion of the total number of pixels in the image. The result obtained is the mesophase pitch content corresponding to this section, which is then converted into volume content to obtain the mesophase pitch content of the asphalt sample corresponding to this stage.

### 3.9. X-ray Diffraction Analysis (XRD)

The crystal structure of the sample was determined using a D/max2550VB/PC X-ray diffractometer. The testing process employed a Cu target with a wavelength (λ) of 0.15406 nm and covered an angular range of 5–75°. The microcrystalline parameters, such as those shown in Formulas (2)–(4), were calculated utilizing the Scheler equation and the Bragg equation:

(4) $${\;d}_{002}=\frac{\lambda }{2sin\theta_{002}}$$

(5) $$L_{c}=\frac{K\lambda }{\beta_{002}cos\theta_{002}}$$

(6) $$\;N=\frac{L_{c}}{d_{002}}+1$$

Herein, θ_002_ represents the diffraction angle of the (002) crystal plane, while d_002_ denotes the interlayer spacing of graphite, measured in nm. The parameter K is a constant associated with the shape of the crystalline grains, typically set as K = 0.94. Lc refers to the average thickness of the crystal’s stacked layers, measured in nm. β_002_ represents the half-peak width of the (002) peak, measured in radians. Lastly, N signifies the average number of layers in the graphite microcrystalline structure.

### 3.10. Time-of-Flight Mass Spectrometry (TOF-MS)

Asphaltene exhibits a wide distribution of molecular weights. Analyzing the average molecular weight and distribution of asphaltene and its co-carbonization products allows for the determination of the peak molecular weight present in asphaltene, thereby aiding in the elucidation of its average molecular structure. The instrument utilized in this study is the 4800 plus time-of-flight mass spectrometer, manufactured by ABS Company in Singapore. This instrument was employed to analyze samples of asphaltene and modified mesophase pitch to investigate their molecular characteristics.

### 3.11. Thermogravimetric Analysis (TG) and Differential Thermal Analysis (DTG)

The thermal weight loss behavior of the sample was investigated using a TGA 8000 thermogravimetric analyzer manufactured from Shanghai branch of Perkin-Elmer Company in the USA. The experimental measurements were performed under a controlled nitrogen atmosphere, with the temperature ramped up from ambient conditions to 800 °C at a constant heating rate of 10 °C/min. By meticulously recording the alterations in sample mass as a function of temperature, both the weight loss curve and weight loss rate curve of the sample were obtained.

### 3.12. Scanning Electron Microscope (SEM) Characterization

The surface topography of modified mesophase pitch samples (MMP-0, MMP-1, MMP-2, MMP-3, and MMP-5) was examined using the FEI Quanta 200FEG field emission environmental scanning electron microscope manufactured by Philips. This instrument, with a resolution of 6.0 nm, an acceleration voltage range of 20–25 KV, and adjustable magnification from 100,000 to 200,000 times, allowed for detailed observation of the sample’s surface morphology. Additionally, the JEOL S4800 scanning electron microscope was utilized to explore the microstructure of the mentioned materials.

### 3.13. Raman Spectroscopy Analysis

The microcrystalline content and arrangement of carbon layers in the sample were characterized using the DXR3xi Raman Imaging Microscope of The East China University of Science and Technology. The main parameters of the instrument are as follows: laser: 785 nm; laser excitation: 50–3500 cm^−1^; 10× objective; and exposure time: 1.0 s. The determination of microcrystalline content and carbon layer arrangement primarily relies on the ratio between the D-band, located near 1350 cm^−1^, and the G-band, positioned at 1625 cm^−1^. I_D_/I_G_ is the ratio of the intensities of the D and G peaks in the Raman spectrum.

## 4. Materials and Methods

### 4.1. Materials

The FCC slurry utilized in this study was obtained from the Baling Petrochemical Plant [24] (in the following text, the FCC slurry will be abbreviated as “FCC BL”). Prior to experimentation, the FCC slurry underwent a deasphaltene treatment process, resulting in a treated FCC-BL sample with an asphaltene content of less than 0.5%. The performance characteristics of the FCC-BL sample are depicted in Table 4. The research team conducted direct thermal condensation treatment on the FCC slurry and analyzed the properties of the mesophase pitch products. The findings revealed that the optimal conditions for producing mesophase pitch from FCC-BL were a temperature range of 400 °C to 420 °C, a pressure range of 1 to 2 MPa, and a reaction time between 4 to 8 h. Some other physical properties of FCC-BL are referred to in Table S1.

### 4.2. Obtaining Co-Carbonizing Agents

A supercritical fluid extraction and fractionation experimental device was employed to separate asphaltene, as illustrated in Figure S1. The experimental setup involved utilizing 500 g of Tahe crude oil as the raw material and n-hexane as the solvent. The extraction and fractionation tower was operated with controlled temperatures, where the top temperature was maintained at approximately 240 °C, with the upper part of the tower at 230 °C, the lower part at 220 °C, and the bottom at 210 °C. Each experimental run involved injecting 500 g to 1000 g of the sample, with a pressure range of 2.0 to 10.0 MPa and a linear pressure increase rate ranging from 0.25 to 2.50 MPa/min. The solvent circulation rate was set between 50 and 150 mL/min. The entire process was conducted under constant pressure conditions, and the final product, asphaltene, was obtained at the specified nozzle. The properties of the obtained asphaltene are summarized in Table 4.

### 4.3. Preparation of Modified Pitch

**Figure 9:**
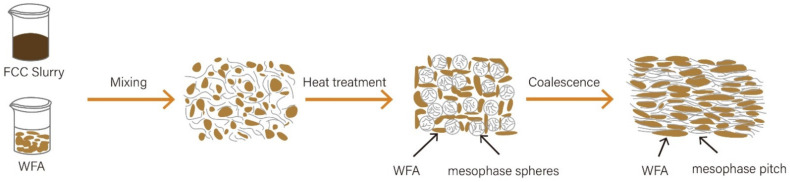
The schematic drawing of the influential mechanism of WFA and FCC-BL in pyrolysis and carbonization.

Upon discharge from the experimental apparatus, the asphaltene appears as a dense black solid. Initially, the asphaltene was subjected to a drying process at room temperature for a minimum of 6 h. Subsequently, the asphaltene was pulverized into a powdered form. Different proportions of asphaltene (0%, 1%, 2%, 3%, and 5%) were then mixed with 50 g of FCC slurry. The mixture was heated to various temperatures (ranging from 400 to 420 °C) and exposed to different reaction times (4 to 8 h) and reaction pressures (ranging from 0 to 2 MPa). The rotational speed of the reaction was maintained between 300 and 500 rad/min. Throughout the reaction, it was necessary to periodically vent the generated gases resulting from the heating process to maintain a constant gas pressure. Once the reaction was complete, the temperature inside the reaction vessel was allowed to naturally cool to room temperature. Subsequently, the co-carbonization reaction products were extracted and subjected to characterization analysis. A picture of the reaction vessel is shown in Figure S2. These samples are designated as MMP-X, where X represents the percentage of asphaltene used and MMP stands for modified mesophase pitch. The reaction mechanism of this experiment is shown in Figure 9 [56,57].

## 5. Conclusions

This study presents a methodology for effectively reutilizing asphaltene and producing high-quality mesophase pitch. The research group employed asphaltene as a modifying agent and utilized co-carbonization techniques to synthesize mesophase pitch. The addition of asphaltene had a discernible impact on the nucleation of the mesophase, resulting in an increase in the formation of mesophase small spheres. Moreover, the introduction of asphaltene during the carbonization process led to an augmentation of alkyl and aromatic hydrocarbons, thereby facilitating the formation of mesophase and enhancing both the quantity and quality of the resulting asphalt. Under specific conditions including a temperature of 420 °C, a pressure of 1 MPa, and a reaction time of 6 h, a mesophase pitch with a satisfactory softening point, broad and extensive texture, high product yield, and well-defined carbon layer structure could be obtained when asphaltene was added at a concentration of 2%. The addition of a small amount of asphaltene was found to facilitate mesophase transformation and improve the degree of optical anisotropy. The presence of a higher number of naphthenic structures in asphaltene exerted a more pronounced hydrogen transfer effect, promoting the generation of mesophase small spheres and the formation of wide-area mesophase pitch. Nevertheless, when the asphaltene content surpassed a certain threshold, it tended to cover the surface of mesophase small spheres, impeding their contact and compromising the optical texture of the resultant mesophase pitch products. Additionally, asphaltene contributed to the enhancement of polymerization reactions within the system, leading to an increased average molecular weight of the mesophase pitch. The addition of 2% asphaltene resulted in the minimum I_D_/I_G_ value for mesophase pitch, indicating superior molecular orientation and larger graphite-like microcrystal size. Furthermore, it was observed that the maximum θ and Lc values and the minimum d_002_ value were achieved when 2% asphaltene was added, suggesting the highest degree of order in the mesophase pitch. The inclusion of asphaltene also elevated the yield and aromaticity of the mesophase pitch, while significantly improving its thermal stability.

## Figures and Tables

**Table 1:** Basic properties of different modified mesophase pitch.

Sample	Elemental Analysis (wt%)		H/C Ratio	Solubility (wt%)		Yield (%)	Softing Point	Ash (%)
	C	H		TI	QI			
MMP-0	89.77	6.86	0.92	23.8	1.3	76.3	243	0.20
MMP-1	90.86	6.27	0.83	30.7	3.2	79.5	267	0.23
MMP-2	91.63	5.59	0.73	29.9	3.7	82.7	289	0.22
MMP-3	92.47	5.23	0.68	35.2	8.6	84.3	307	0.26
MMP-5	94.45	4.68	0.59	40.3	17.8	89.2	344	0.25

**Table 2:** Microcrystalline parameters of different modified mesophase pitches.

Sample	XRD				Raman
	2θ/Degree	d_002_/nm	L_c_/nm	N	I_D_/I_G_
**MMP-0**	26.003	0.3424	1.910	6.578	1.03
**MMP-1**	26.216	0.3396	2.008	6.913	0.98
**MMP-2**	26.380	0.3376	2.228	7.599	0.92
**MMP-3**	26.277	0.3389	1.973	6.822	1.02
**MMP-5**	25.919	0.3434	1.881	6.478	1.06

**Table 3:** Proton distribution of the different modified mesophase pitches determined by ^1^H-NMR and ^13^C-NMR analysis.

Sample	^1^H-NMR (%)									^13^C-NMR (%)		
	H_ar(mono)_	H_ar(di)_	H_a(poly)_	H_ar_	H_α_	H_β_	H_γ_	H_al_	H_ar_/H_al_	C_sat_	C_ar_	C_ar_/C_sat_
MMP-0	6.45	40.65	5.17	52.27	27.53	17.34	2.86	47.73	1.095	14.29	85.71	5.998
MMP-1	6.09	41.28	7.75	55.12	30.68	12.45	1.75	44.88	1.228	13.17	86.83	6.593
MMP-2	6.99	42.98	9.48	59.45	30.61	8.71	1.23	40.55	1.466	11.89	88.11	7.410
MMP-3	4.94	44.18	13.21	62.33	31.88	5.23	0.56	37.67	1.655	9.27	90.73	9.787
MMP-5	4.92	45.52	17.71	68.15	28.92	2.79	0.14	31.85	2.140	7.14	92.86	13.006

**Table 4:** Basic properties of the experimental materials.

Material	Elemental Analysis (wt%)					H/C Ratio	Solubility (wt%)		Softing Point	Ash (wt%)
	C	H	N	O	S		TI	QI		
FCC-BL	90.55	7.66	1.02	0.32	0.45	1.02	<0.1	<0.1	20	0.02
WFA	86.20	8.46	1.21	1.80	2.33	1.18	22.5	1.6	145	0.53

TI: Toluene insoluble substance; QI: quinoline insoluble substance.

## Data Availability

Data are contained within the article and Supplementary Materials.

## Supplementary Materials

The following supporting information can be downloaded at: https://www.mdpi.com/article/10.3390/molecules29071500/s1. Figure S1. Schematic diagram of asphaltene separation equipment; Figure S2. Schematic diagram of carbonization; Figure S3. Optical structure of modified mesophase pitch under different reaction temperature; Figure S4. Optical structure of modified mesophase pitch under different reaction time; Figure S5. Optical structure of modified mesophase pitch under different reaction pressures; Table S1. Composition and properties of FCC-BL; Table S2. Classification of the optical microstructure of mesophase pitch; Table S3. The FT-IR characteristic absorption peaks of common structural; Table S4. The proton chemical shift in the 1H-NMR.
