# Supplementary material for: The Neglected Role of Asphaltene in the Synthesis of Mesophase Pitch
Source: Molecules. 2024 Mar 27;29(7):1500. doi: 10.3390/molecules29071500 (PMC11013708; doi:10.3390/molecules29071500)
Supplement: Supplementary file 1 [file molecules-29-01500-s001.zip › molecules-2902484-supplementary.pdf]

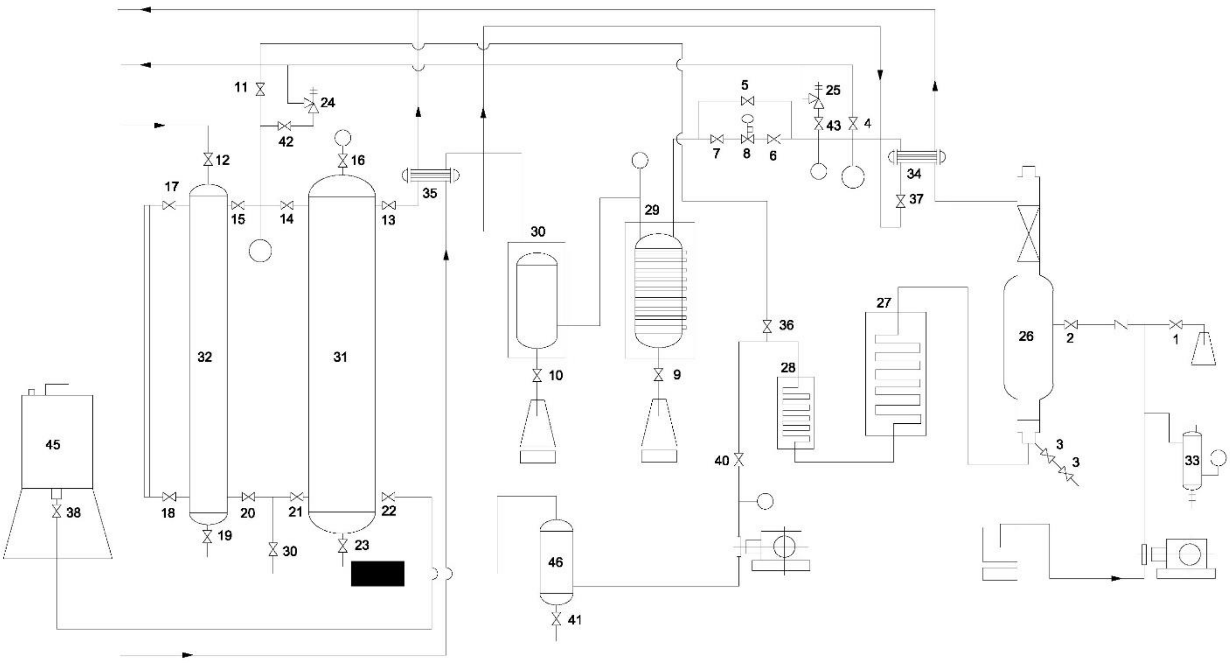

Figure S1. Schematic diagram of asphaltene separation equipment.

1. Drain valve of slag pump; 2. Raw material inlet valve; 3. Extraction kettle discharge valve; 4 high pressure vent valve; 5. Emergency bypass valve; 6. Cut off valve 1; 7. Cut off valve 2; 8 Automatic regulating valve; 9. Separator lofting valve; 10 redistributor lofting valve; 11 Low pressure vent valve; 12. Nitrogen pressure valve; 13. Solvent circulation valve; 14. Solvent tank connecting valve; 15. Solvent tank connecting valve; 16 solvent tank pressure valve; 17. Liquid level pipe valve 1; 18. Liquid level pipe valve II; 19. Small solvent tank emptying valve; 20. Solvent tank connecting valve; 21. Solvent tank connecting valve; 22. Solvent refill valve; 23. Large solvent tank emptying valve; 24. Low pressure safety valve; 25. High pressure safety valve; 26. Extraction kettle; 27. Two-stage solvent preheater; 28. Solvent preheater; 29. Separator; 30. Redistributor; 31. Large volume tank; 32. Small volume tank; 33. Pressure gauge isolation tank; 34. Straight tube condenser; 35. Coil condenser; 36. Solvent pump vent valve; 37. Straight tube condenser water inlet valve; 38. Solvent refill tank outlet valve; 39. Solvent pump inlet valve; 40. Solvent pump outlet valve; 41. Solvent buffer tank emptying valve; 42. Low pressure safety valve inlet valve; 43. High pressure safety valve inlet valve; 44. Pressure gauge height tank vent valve; 45. Liquid solvent refill tank; 46. Solvent oxygen flushing tank;

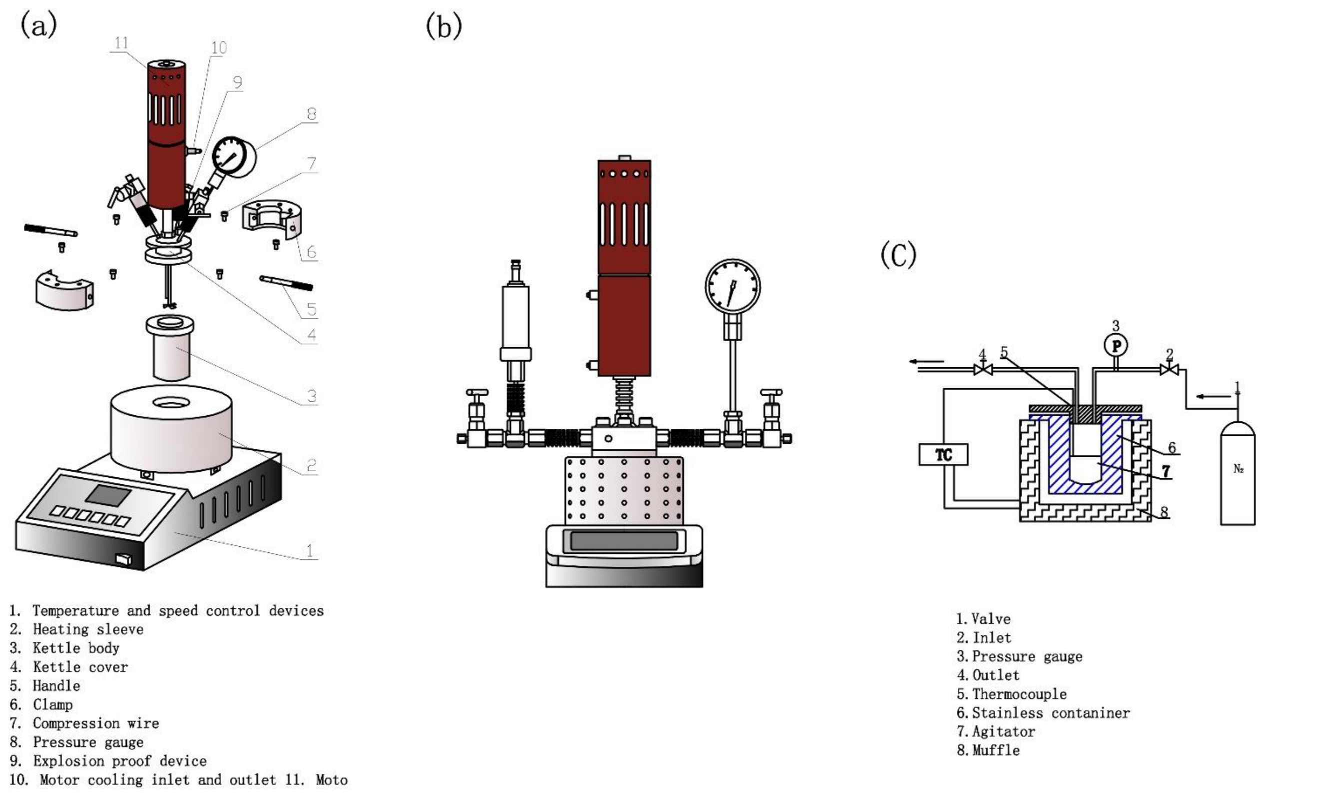

Figure S2. Schematic diagram of carbonization equipment.

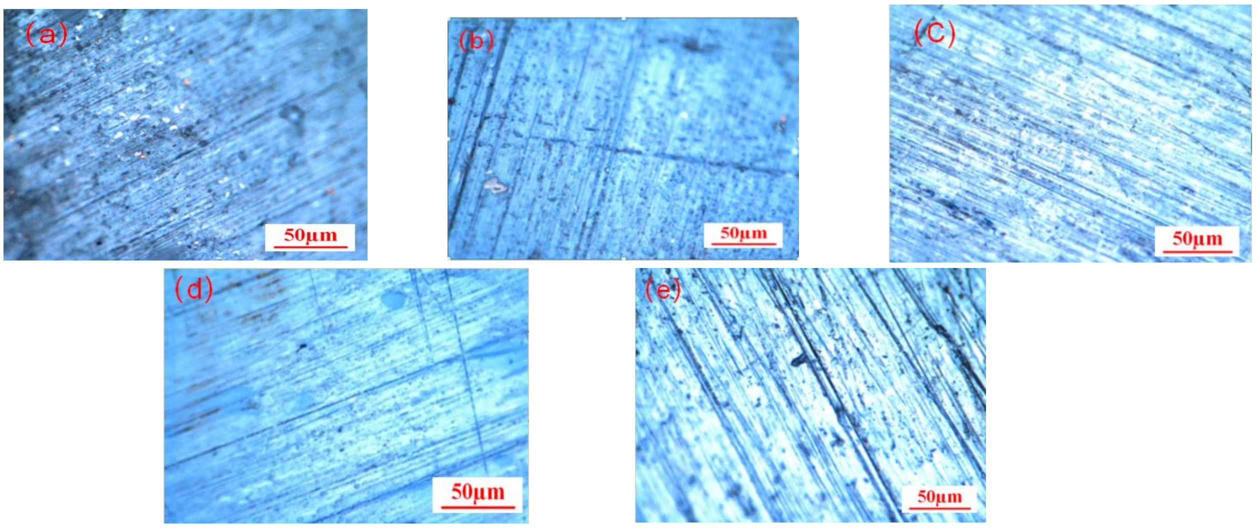

（a）390℃；（b）400℃；（c）410℃；（d）420℃；（e）430℃；

Figure S3. Optical structure of modified mesophase pitch under different reaction temperature.

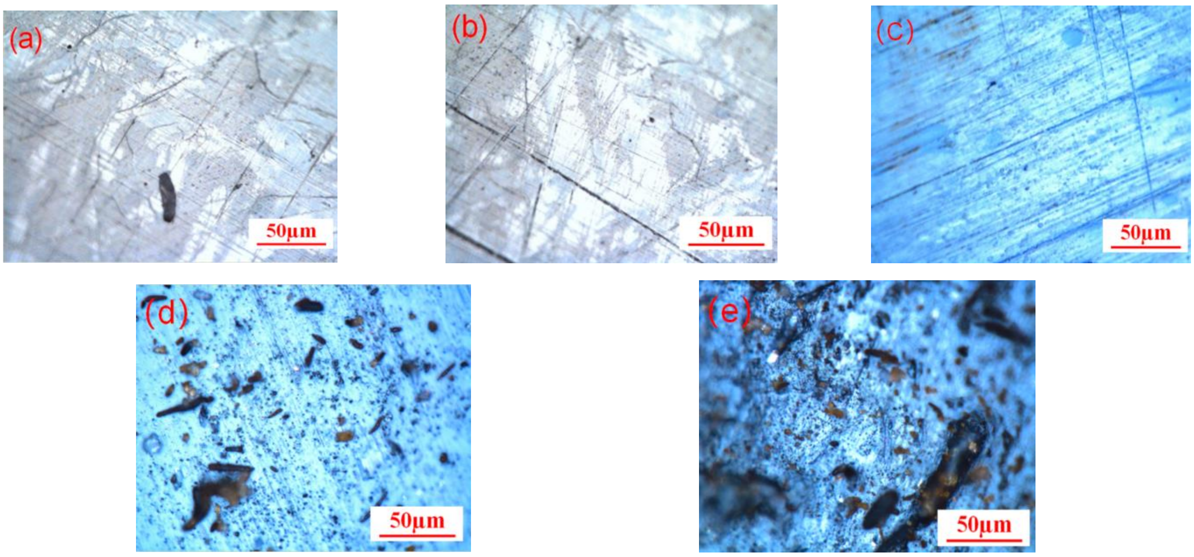

（a）4h；　（b）5h；　（c）6h；　（d）7h；　（e）8h；

Figure S4. Optical structure of modified mesophase pitch under different reaction time.

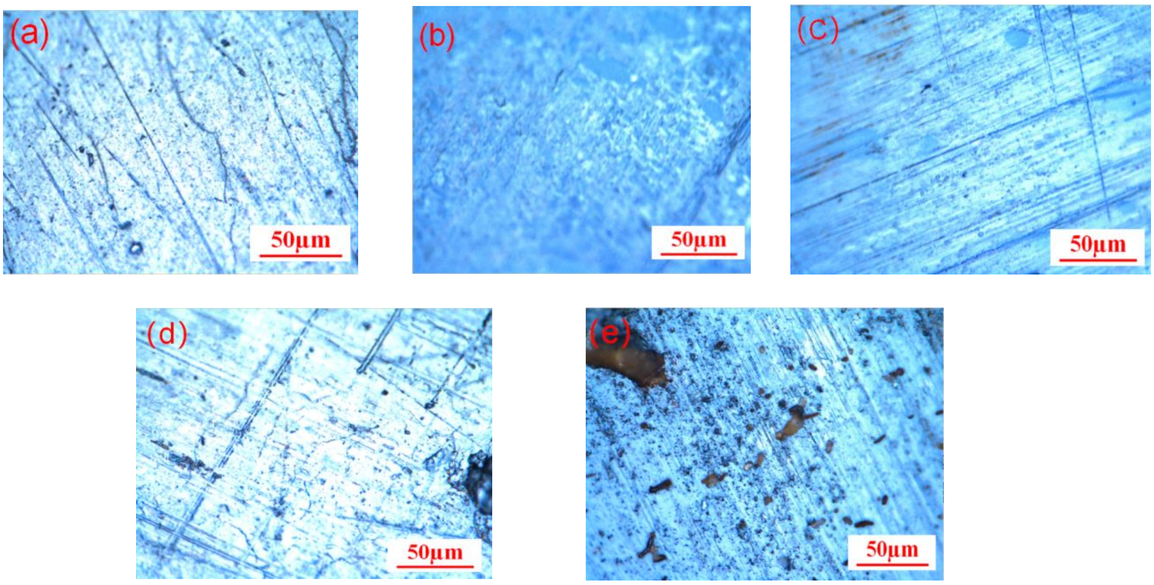

（a）0 MPa；（b）0.5 MPa；（c）1.0 MPa；（d）1.5 MPa；（e）2.0 MPa；

Figure S5. Optical structure of modified mesophase pitch under different reaction pressures.

Table S1. Composition and properties of FCC-BL.

Items	Properties	FCC-BL
Selected properties	Density（25℃，g/cm^3）	1.0476
	Viscosity（80℃，mm^2/s）	37.42
	Molecular weight	486.8
	Ash（μg/g）	140
	Residual carbon（wt %）	8.3
SARA fractions analysis	Residual carbon（wt %）	7.39
	Aromatics（wt %）	80.11
	Resins（wt %）	12.08
	Asphaltenes（wt %）	0.42
Elemental analysis	C（wt %）	90.55
	H（wt %）	7.66
	N（wt %）	1.02
	S（wt %）	0.45
	O（wt %）	0.32
	H/C	1.02

Table S2. Classification of the optical microstructure of mesophase pitch.

Anisotropic optical texture	Size/[μm]	
	Length	Width
Coarse flaky texture	>30	>30
Fine flaky texture	10-30	10-30
Mosaics texture	≤10	<10
Fine fiber texture	<30	<10
Coarse fiber texture	>30	>10

Table S3. The FT-IR characteristic absorption peaks of common structural.

Wavenumber (cm^{-1})	Aliphatic and aromatic groups
3450-3400	-OH stretch
3050-3030	Aromatic CH stretch
2970-2850	Aliphatic CH_3, CH_2 and CH stretch
1775-1765	C=O stretch in ester with group attached to single-bonded oxygen
1735	C=O stretch in ester
1720-1690	C=O stretch in ketone, aldehyde, and carboxyl
1650-1630	C=O stretch, highly conjugated
1600	Aromatic C=C ring stretch, highly conjugated hydrogen-bonded C=O stretch
1510	Aromatic C=C ring stretch
1460-1440	Aliphatic chains CH_3-and CH_2-
1365-1355	Aliphatic chains CH_3- or in-plane O-H bend
1275-1200	C-O-C stretch of alkyl aryl ethers, ester CC(=O)-O stretch and C-O stretch in phenols
1115-1110	C-O-C stretch of aliphatic ethers or C-Ostretch in alcohols
1030	C-O stretch of aryl alkvl ethers or C-Ostretch in alcohols
900-700	Aromatic CH
860	Isolated aromatic H
833 (weak)	1,4-Substituted aromatic groups
815	Isolated Hand/or two neighboring H
750	1,2-Substituted, i.e., four neighboring H

Table S4. The proton chemical shift in the ^{1}H-NMR.

Hydrogen type	Chenmical shift/ppm	Affiliation
H_{ar}	9.0-6.0	Aromatic hydrogen
H_α	4.5-2.0	Aliphatic hydrogens of naphthenic rings or methylene groups on ax-position to aromatic ring
H_β	2.0-1.1	Aliphatic hydrogens of methylene groups on β-position and further from aromatic ring
H_γ	1.1-0.3	Aliphatic hydrogens in methyl groups in γ-position to an aromatic ring
H_n	2.0-1.4	Naphthenic hydrogen
